# Isolation and Enrichment of Circulating Fetal Cells for NIPD: An Overview

**DOI:** 10.3390/diagnostics11122239

**Published:** 2021-11-30

**Authors:** Giulia Sabbatinelli, Donatella Fantasia, Chiara Palka, Elisena Morizio, Melissa Alfonsi, Giuseppe Calabrese

**Affiliations:** 1 Dipartimento di Neuroscienze, Imaging & Scienze Cliniche, Scuola Superiore G. D’Annunzio, University of Chieti, 66100 Chieti, Italy; giulia.sabbatinelli@unich.it; 2 UOSD Genetica Oncoematologica, Dipartimento di Oncologico-Ematologico, Ospedale Spirito Santo, ASL Pescara, 65124 Pescara, Italy; donatella.fantasia@asl.pe.it; 3 UOC Genetica Medica, Ospedale S.S. Annunziata, ASL2 Chieti, 66100 Chieti, Italy; chiara.palka@asl2abruzzo.it (C.P.); melissa.alfonsi@gmail.com (M.A.); 4 Genetica Medica, Dipartimento di Tecnologie Avanzate in Medicina e Odontoiatria, School of Medicine, University of Chieti, 66100 Chieti, Italy; elisena.morizio@unich.it

**Keywords:** prenatal diagnosis, circulating fetal cells, NIPD

## Abstract

Prenatal diagnosis plays a crucial role in clinical genetics. Non-invasive prenatal diagnosis using fetal cells circulating in maternal peripheral blood has become the goal of prenatal diagnosis, to obtain complete fetal genetic information and avoid risks to mother and fetus. The development of high-efficiency separation technologies is necessary to obtain the scarce fetal cells from the maternal circulation. Over the years, multiple approaches have been applied, including choice of the ideal cell targets, different cell recovering technologies, and refined cell isolation yield procedures. In order to provide a useful tool and to give insights about limitations and advantages of the technologies available today, we review the genetic research on the creation and validation of non-invasive prenatal diagnostic testing protocols based on the rare and labile circulating fetal cells during pregnancy.

## 1. Introduction

During the early stages of fetal development, some cells migrate from the fetus to the maternal circulation and may represent an interesting target for non-invasive prenatal diagnosis (NIPD).

However, despite technological advances, an effective standardized test suitable for routine clinical practice is not yet available. An obstacle is the small number of fetal cells present in the maternal circulation and their extreme fragility, which can lead to loss during sample handling and the absence of a specific fetal marker.

Non-invasive prenatal testing based on circulating fetal cell-free DNA has been commercially available since 2011 and offers the opportunity to radically change prenatal screening. However, the introduction into actual clinical practice is challenging because of cost, differences in the scope of abnormalities detectable, and integration into existing testing [1,2]. Recently, the analysis of fetal cells from peripheral maternal blood has been shown to be more effective in helping to identify fetal aneuploidy, microdeletion syndromes, hemoglobinopathy, and blood groups than cffDNA, due to their intact fetal genome, free from maternal DNA contamination [3]. 

In this review, we briefly retrace the steps of this long journey, which has been characterized by failures and technological progress and has lasted decades.

## 2. Limitations and Difficulties

The great potential of circulating fetal cells in non-invasive prenatal testing was identified in 1969 by Walknowska et al., with the discovery of fetal lymphocytes with a male karyotype in the maternal circulation [4], even though the discovery of fetal cells is attributed to Zipursky et al., 10 years earlier [5].

This started the chapter on the study of circulating fetal cells. The greatest limitations have been the small number of fetal cells, their dubious origin, and the difficulty of developing an adequate isolation strategy due to the lack of specific markers and the fragility of the fetal cells.

A variety of fetal cell types, such as trophoblastic cells, lymphocytes, hematopoietic stem cells (HMCs), and fetal nucleated erythrocytes (fnRBCs), can be found in the maternal circulation [6]. However, it is essential to remember that ideal target cells for non-invasive investigation need to express characteristics that demonstrate their fetal origin, such as short half-life, early appearance in the maternal circulation, relatively distinct cell morphology, a single nucleus with complete genetic makeup, or specific cellular markers. The fetal cell lines found in the mother’s blood have different characteristics, and the biggest obstacle is the separation of these fetal cells from those of the mother; this can significantly influence the accuracy of investigations.

### Quantification of Fetal Cells in Maternal Blood

The entry of fetal cells into the maternal circulation is a physiological phenomenon called microchimerism, which occurs as early as 4–6 weeks into pregnancy. Fetal cells have been detected in maternal blood from 4 weeks of gestation until 39 days after delivery. The optimal time for recovery remains to be determined [7,8,9,10,11,12,13,14]. The total number of fetal cells in maternal blood was comprehensively verified in 2001 by Krabchi et al. via a method that avoided the cell enrichment of blood samples, but using molecular cytogenetic techniques such as fluorescence in situ hybridization (FISH) and primed in situ labeling (PRINS). Krabchi et al. found that there are 2–6 fetal cells/mL of maternal blood in the second trimester of a normal pregnancy [15]. However, fetal and placental abnormalities, such as fetal aneuploidy, preeclampsia, maternal-fetal isoimmunization, and other maternal obstetric complications such as gestational diabetes and bleeding, result in an increase in the number of fetal cells of different types in the maternal circulation [16,17,18]. 

Numerous studies have verified this condition. In particular, according to Krabchi, in the case of an aneuploid fetus, the number of fetal cells is approximately 6–32 per mL of maternal blood (1 fetal cell for every 200/250 maternal cells after enrichment), considering all the fetal cells present and independent of their morphology. This condition does not currently have a clear explanation but is supposed to depend on a modification of the microscopic architecture of the placenta. Alternatively, it may be related to the size of fetal cells, which differs from those of cytogenetically normal fetuses of the same gestational age [18,19].

## 3. Fetal Cell Types and Strategies for Isolation and Enrichment

Herzenberg et al. were the first to demonstrate the enrichment of fetal leukocytes from maternal blood in 1979 using fluorescence-activated cell sorting (FACS) [20]; however, the most studied cell types are erythroblasts (nRBCs) and trophoblasts. A summary of the types of fetal cells identified and isolated from the maternal circulation and the most commonly applied markers are shown in Table 1.

### 3.1. Trophoblast

Trophoblasts were the first cell type identified. Cytotrophoblast-derived cells are mononuclear, invade the uterine wall and spiral arteries, and spill into maternal blood.

One of the distinguishing features of trophoblastic cells is the expression of cytokeratins (CKs).

Sargent et al. and Johnson et al. used an antibody against trophoblast-specific H315 surface antigen, which later proved insufficient for adequate cell separation [44,46,47]. 

Cacheux et al. recovered trophoblasts from maternal blood using immunomagnetic leukocyte depletion and flow cytometry with the anti-trophoblast antibodies GB17 and GB25. However, Y chromosome identification by FISH showed a rate of only 4.25% [45]. Other studies report the use of human leukocyte G antigen to enrich circulating trophoblasts from the maternal circulation [37,38,42,50]. An additional isolation strategy was based on fetal trophoblast cell size [55]. 

The limitations currently associated with the use of these cells are their extremely low numbers in the mother’s blood. In addition, they are difficult to isolate because they become trapped in the lungs and are rapidly eliminated from the maternal circulation. Furthermore, the passage of trophoblastic cells does not appear to be a common phenomenon in all pregnancies [52,53,54].

The major disadvantage of using CKs and other intracellular antigens is the need to make membranes of fragile fetal cells permeable to antibody molecules.

In addition, trophoblastic cells, being of placental origin, have a 1% incidence of mosaicism, similar to that found in chorionic villus sampling (CVS) [55].

### 3.2. Erythroblast

Historically, nRBCs have been the most studied cells for NIPD. The first evidence of immature erythrocytes circulating in maternal blood dates back to 1957 in Kleinhauer’s studies [36]. The fnRBCs are the first hematopoietic cells to be produced during fetal development. Their passage through the maternal-placental interface is predominant over other fetal cell types, such as leukocytes and trophoblasts. They also have a short half-life (25–35 days), which does not allow their persistence in the maternal circulation [56,57]. In addition, they have a single nucleus with a complete genetic makeup and relatively specific intracellular and surface markers. Due to these characteristics, fnRBCs appear highly interesting targets for NIPD [6].

## 4. Technical Approaches for Isolation or Enrichment of Fetal Cells

### 4.1. MACS and FACS

In the 1990s, the techniques of choice for fnRBC enrichment were FACS and magnetic activated cell sorting (MACS). Bianchi et al. first used a monoclonal antibody against CD71 to enrich nRBCs [58]; selection by only transferrin receptor recognition was subsequently used by Ganshirt-Ahlert et al. [34], but this antibody also recognizes some adult cells. To improve the purity, Purwosunu et al. added the depletion of maternal cells and fetal lymphocytes with anti-CD45 antibody prior to positive selection for CD71 [54]. Many different positive and negative enrichment strategies have been explored, including positive enrichment by MACS with monoclonal antibodies to CD71 or glycophorin A (Gly A) or detection by FACS using monoclonal antibodies to fetal hemoglobin (HbF). Some examples are the work of Martel-Petit et al., who identified a fetus with cystic fibrosis by studying fnRBCs [39], and Souza et al., who used a combination of antibodies to increase the specificity of fnRBC selection [40].

Studies have shown that the best results are obtained with magnetically based separation systems as compared to flow-sorting [17]. However, the recovery rate of fnRBCs is low for clinical implementation because the key to the enrichment of cell populations by FACS and MACS is the selectivity of the antibody [59].

### 4.2. Density Gradient Centrifugation

The fnRBCs have a density higher than that of white blood cells. The most commonly used gradients have been made using Ficoll and Percoll at different densities (1.077 g/mL, 1.083 g/mL, 1.119 g/mL). In this case, the buffers that are used have different cell recovery rates. Through this approach, up to 1000-fold enrichment in the fetal cell population can be achieved, but there is still significant contamination with maternal cells [11]. Furthermore, it appears that the relative density of fetal cells can change during the preparation of blood samples, making it difficult to standardize density gradient centrifugation procedures.

Sitar et al. used non-physiological conditions to modify the cell mean corpuscular volume of red blood cells and increase mononuclear cell densities. To overcome the drawbacks inherent in a standard centrifuge tube, they used a cell separation device to isolate fetal cells in a single density gradient centrifugation [60]. The enrichment by centrifugation on a density gradient was subsequently combined with the application of antibodies for positive selection of fnRBCs. Our group used an anti i-antigen antibody in combination with antibodies for CD71+, CD45−, and CD19− cells on samples previously enriched by centrifugation on a density gradient. Positive fnRBCs were recovered by micromanipulation [38]. The blood group I-antigen is a surface marker of great interest for the identification of fnRBCs. Adult human red blood cells fully express I-antigens and contain only a few i-antigens, whereas fetal and neonatal red blood cells predominantly express the latter [61]. After birth, i-antigen is gradually replaced by I-antigen, but this may not occur if the subject has thalassemia major, hypoplasia anemia, or acute leukemia [62,63]. 

We also performed a clinical feasibility study on 172 women with a single pregnancy prior to any invasive prenatal diagnostic (IPD) procedure to investigate the presence of fetal aneuploidies. Fetal cell enrichment was performed by transfer under non-physiological conditions and density gradient centrifugation. Trisomic fetal cells were identified by dual-probe FISH analysis [61]. Seven of the samples exhibited fetal aneuploidy, which was confirmed by invasive prenatal diagnostic procedures. After enrichment of fetal cells, the frequency of trisomic cells was at least double in samples from aneuploid pregnancies (range 0.38–0.90%) compared to samples from normal pregnancies (≤0.18%; *p* < 0.01). One false-negative result was also obtained. Overall, the FISH analysis had a detection rate of 7/8 (87.5%, 95% CI 42–99%) [64]. In a subsequent study, we applied the same fetal cell enrichment approach to a cohort of pregnant women at high risk based on a contingent test. FISH analysis identified 7 abnormal and 39 normal cases, with 100% specificity and 100% sensitivity [65]. In a recent study, Nemescu et al. combined double-density gradient centrifugation with paramagnetic selection by the conversion of hemoglobin (Hb) into methemoglobin [41]. The advantage of the study was a simple approach with low cell manipulation, and the suggestive results from a limited number of isolated fetal cells analyzed need further investigation on a large cohort of patients.

### 4.3. Other Methods

Other methods used for fetal cell enrichment include avidin-biotin columns, magnetic iron-fluid, and lectin-induced separation. To improve enrichment, some researchers have sought more specific antibodies or biochemical markers, whereas some have relied on chemical assays for analysis, such as 2,3-bisphosphoglycerate (BPG) [66], carbonic anhydrase (CA) [67,68], and thymidine kinase (TK) [69]. It is thought that, in a sequential peroxidase reaction, BPG exposes fetal heme-iron to oxidation by identifying HbF by forming a colored substrate-associated complex. In addition, fetal cells can be differentiated from adult cells based on TK enzyme activity, which is virtually absent in adult cells, using a fluorescent analogue of thymidine. The fnRBCs are also less susceptible to ammonium chloride lysis than adult nRBCs because CA activity is five times lower and acetozolamide permeability approximately ten times greater. Several studies [70,71] have used soybean agglutinin (SBA), which recognizes N-acetylgalactosamine and galactose, demonstrating high specificity for erythroblasts and eliminating leukocytes from the sample.

However, lectin-induced separation should be combined with another enrichment technique, usually density gradient centrifugation.

The possibility of using an erythropoietin assay has also been explored [72]. However, to date, most researchers still use anti-CD71 or anti-globin antibodies for the enrichment of fnRBCs.

Other researchers have isolated a fair number of nRBCs by charge flow separation, which allows cells to be sorted according to their characteristic surface charge densities [73,74].

## 5. New Approaches

In recent years, numerous studies have reported the implementation of technology in the field of microfluidics. In fetal cell isolation, microfluidics allows the separation of cells based on size, deformability, and electrical and optical properties [75,76]. Antibodies with specific cellular markers can also be used [77]. Wang et al. were able to isolate 24 fnRBCs per milliliter of maternal blood in media using gelatin-coated microspheres, with anti-CD147 as a specific recognition molecule. Cells that bind to the microspheres are separated through a spiral microfluidic chip, based on the size difference between the microspheres binding fnRBCs and white blood cells. The release of fetal cells from the microspheres is achieved by enzymatic treatment. A fetal cell capture efficiency of approximately 81%, a purity of 83%, and a viability of cell release >80% were calculated using spiked samples [78]. The technology is very suggestive, and robust evidence is needed about its practical application when tested on a large series of patients. 

Another fascinating technological innovation is the Cell Reveal^TM^ system, a silicon-based nanostructured microfluidic that uses immunoaffinity to capture trophoblasts and fnRBCs with specific antibodies. Huang et al. tested this technology, isolating 14–32 fnRBCs/4 mL and 1–44 EVT/4 mL of maternal blood. The fetal origin of the cells was confirmed by FISH on chip. The identified cells were retrieved by an automated cell harvester for molecular genetic analysis, such as comparative genomic array hybridization (aCGH) and next-generation sequencing (NGS) [79]. The cell capture rate was evaluated in spiking assays and estimated to be 88.1% [80].

In a recent study, Gur et al. presented a two-tiered microchip system that reduces sample preparation steps while implementing purity, using ETVs model cell lines. The system allows for direct processing of a whole blood sample and appears versatile enough to target a variety of antigens to achieve a high rate of cell recovery [81].

Microfluidic techniques are already used in many laboratories because of the considerable advantages they present, all of which are derived from the small volumes of liquid treated, high control of flow, less time needed to analyze a product, lower costs of reagents and waste products, greater control of concentrations and molecular interactions, and the possibility of performing parallel processes. Further development of such devices appears to be very promising.

However, limitations of approaches based on microfluidic systems remain, including the inability to directly process a standard tube of blood sample without an initial volume reduction step, the use of a single antibody for cell capture, and the retrieval of cells individually for downstream analysis [82]. In Table 2 a list of approaches for fetal cell isolation/enrichment is reported.

## 6. Conclusions

Fetal cells, although rare, circulate in the blood of pregnant women in variable numbers. Although we have not yet developed a test that allows for routine clinical isolation of fetal cells, it is possible to enrich and analyze them to determine the blood types, the presence of hemoglobinopathy, and the genetic status of the fetus. Over the years, different strategies for isolating fetal cells have been tested. However, invasive methods, such as CVS and amniocentesis, are still the most reliable methods for prenatal diagnosis of chromosomal aberrations and copy number variations (CNVs). However, these invasive procedures carry a risk of miscarriage and cause fear and stress in the pregnant woman.

With technological progress, it has become possible to recover, identify, and genetically analyze even a few circulating fetal cells from a few milliliters of maternal blood. Whole genome amplification (WGA) allows the use of rare fetal cells for downstream genetic analysis, such as aCGH or NGS. In the near future, NIPD using circulating fetal cells could enter the diagnostic scenario, leading to long-awaited change.

## Figures and Tables

**Table 1:** Fetal cell lines isolated from maternal peripheral blood and markers most commonly applied for validation of NIPD.

Fetal Cell Types in Maternal Blood	Markers	Advantages	Drawbacks
Lymphoid progenitors	CD34 [21,22] CD38 [21,22]	In vitro proliferation [23]	Long-term survival [21,22]; Not easy distinguishable from maternal cells [23]
Hematopoietic stem/progenitor cells	CD34 [6,21,24,25,26,27,28]	In vitro proliferation [23]	Long-term survival [21]; Not easy distinguishable from maternal cells [23]
Mesenchymal stem cells	Vimentin [25,29] Fibronectin [25] Vascular cell adhesion molecule [25,28] CD14 [25,29] CD45 [25,29]	In vitro proliferation and differentiation [25,29,30]; Great therapeutical potential [25]	Very low number [25,29]; Engraftment in maternal tissue soon after transplacental passage [29]
Leukocytes	HLA antigens [20,28,31,32] CD45 [28,33]	Transplacental passage [4]; In vitro proliferation [4]	Long persistence in maternal blood [21]
Nucleated red blood cells	ζ and ε chain of embryonic Hb [34,35,36,37] γ chain of HbF [36] CD71 [38,39,40,41] 4B9 [42] i-antigen [38] CD147 [43] Gly A [39]	Short half-life [6]; Single nucleus [6]; Early appearance in maternal blood [6]; Surface and intracellular markers [34,36,37,38,39,41,42,43]	Low number in maternal blood [6]
Extravillous cytotrophoblasts (EVTs)	H315 [44] GB17 [45] GB25 [45] Cytokeratins [46,47,48] CD105 [48] Human leukocyte G antigen [49,50] CD141 [51]	Specific intracellular markers [52]; Relatively distinctive cell morphology (size) [53]	Passage in maternal blood is uncommon phenomenon in all pregnancies [52,53,54]; Placental origin (1% mosaicism) [55]

**Table 2:** A summary of technical approaches of isolation or enrichment of fetal cells in the maternal circulation.

Fetal Cell Type	Isolation/Enrichment Technique	Purity and Recovery Rates	References
fnRBCs	MACS	N/A	[34,41,83]
	FACS	N/A	[58]
	Density gradient centrifugation	82 ± 6.4% purity [51] 84 ± 4% recovery rate [51] 32.5 ± 11.6 recovered cells [61]	[60,64,65,66,71]
	High molecular filter method	46.3 ± 25.1 recovered cells	[71]
	Morphology-based micromanipulation	Average 4.1 recovered cells	[84]
	Charge flow separation	0.503 ± 0.264 purity 11,409 ± 7.684 recovered cells	[73]
	Lateral magnetophoretic microseparator	87.8% purity 1–396 recovered cells	[85]
	Microfluidics	83% purity [68] 81% recovery rate [68] 88.17% recovery rate [70]	[78,79,80]
	Hyperaggregation	N/A	[86]
Trophoblasts	MACS	N/A	[51]
	FACS	N/A	[45]
	Isolation by size of epithelial tumor/trophoblastic cells (ISET)	50.7% recovery rate [76] 48.1% recovery rate [77]	[50,87,88,89]
	Trophoblast retrieval and isolation from the cervix (TRIC)	99% purity [90] 106% ± 13 recovery rate [90] 675 (IQR, 399–1010) trophoblast yield [91] 97.9% purity [91]	[90,91]
	Microfluidics	1–32 EVT/2 mL	[79]
Fetal cells *	Avidin-biotin immunoaffinity column	Enrichment up to 1000-fold	[92]
	Telomerase depletion assay	N/A	[93]
	data Lectin-based method	7.8 ± 8.5 recovered cells	[70]
	Ikonoscope	N/A	[94]

N/A; not available. * not otherwise identified.
